# Laser enucleation of the prostate in men with very large glands ≥175 ml: A systematic review

**DOI:** 10.1016/j.amsu.2022.104279

**Published:** 2022-07-31

**Authors:** Mehmet Yilmaz, Mustafa Karaaslan, Halil Cagri Aybal, Maximilian Ferry von Bargen, Senol Tonyali, Tuncay Toprak, Christian Gratzke, Arkadiusz Miernik

**Affiliations:** a University of Freiburg – Medical Center, Faculty of Medicine, Department of Urology, Hugstetter Str. 55, 79106, Freiburg, Germany; b Bingol State Hospital, Department of Urology, Bingol, Turkey; c Ankara Polatlı Duatepe State Hospital, Department of Urology, Ankara, Turkey; d Istanbul University Istanbul School of Medicine, Department of Urology, Istanbul, Turkey; e University of Health Sciences, Fatih Sultan Mehmet Training and Research Hospital, Department of Urology, Istanbul, Turkey

**Keywords:** Giant prostate, Laser enucleation, Prostatectomy, Very large prostate

## Abstract

**Background:**

Surgical treatment options for lower urinary tract symptoms can differ according to prostate size. There are few studies on the efficacy and safety of endoscopic enucleation of prostate (EEP) in patients with very large prostates focusing on laser as energy source. In this systematic review, we aimed to examine the efficacy and safety of laser-based EEP on prostate glands ≥150 ml.

**Methods:**

A systematic search was conducted using Web of Science, PubMed-MEDLINE, Wiley Online Library and Cochrane Library databases with the following search terms solely or in combination: "large prostate", "laser enucleation", "laser prostatectomy"by combining PICO (population, intervention, comparison, and outcome) terms. Preferred Reporting Items for Systematic Reviews and Meta-analysis guidelines were followed.

**Results:**

We retrieved 6 studies included 375 patients with prostate sizes ≥175 ml treated with laser-based EEP for symptomatic benign prostatic obstruction. Three studies examined Holmium laser enucleation of prostate (HoLEP) outcomes with a prostate volume (PV) >200 ml, one evaluated HoLEP outcomes with a PV of 200–299 and ≥ 300 ml, two studies evaluated HoLEP outcomes with a PV > 175 ml. We observed improvement in postoperative functional outcomes in patients with a PV > 175, >200 and >300 ml. The retreatment rate was 0–1.3% in all studies involving prostate size ≥175 ml. Most of the complications were Clavien-Dindo I (%0–9) and II (%12.7–16.6).

**Conclusions:**

Laser-based EEP is an efficient, safe and feasible procedure even in very large prostates with good functional outcomes, low perioperative complication and retreatment rates.

## Highlights

• We observed better postoperative functional outcomes in prostates with a volume of ≥175, >200 and >300 ml in the present study.
• The retreatment rate was 0–1.3% in all studies involving prostate size ≥175 ml.
• Laser-based- endoscopic enucleation of the prostate is an efficient, safe and feasible procedure even in very large glands.

## 1 Introduction

Lower urinary tract symptoms (LUTS) due to bladder outlet obstruction (BOO) caused by benign prostatic hyperplasia (BPH) is one of the most frequent health problems impairing adult men's quality of life [1]. Various medical and surgical methods are used to treat LUTS [2]. Thanks to recent technological developments in medicine, the paradigm of surgically treating LUTS has changed. With the popularity of laser use insurgical BPH treatment, laser-based endoscopic enucleation of the prostate (EEP) has come to the fore as an alternative surgical method for transurethral resection of the prostate (TURP) and simple prostatectomy (SP). In particular, Holmium Laser Enucleation of the Prostate (HoLEP) has proven to yield clinical outcomes resembling traditional methods such as TURP and SP [[3], [4], [5], [6], [7], [8], [9]].

Surgical treatment options for BPH may differ according to the prostate's size [2]. Although there is evidence that EEP is feasible regardless of prostate size [10,11], the European Association of Urology (EAU) and American Urological Association (AUA) guidelines recommend laser-based EEP such as HoLEP for prostates with a size >80 ml [12]. In the literature, prostate sizes above 80–100 ml were defined as ‘large prostates’ and most studies on EEP are limited to these prostate sizes [[13], [14], [15], [16], [17]]. However, in clinical reality, prostate sizes exceeding not just the range of 80–100 ml, but also 150 ml, 175 ml and even 200 ml are encountered in considerable numbers [[18], [19], [20], [21], [22], [23], [24], [25]]. The question whether such extremely large glands (>150 ml) should undergo EEP does not seem to be answered. As there are so few investigations on the efficacy and safety of laser-based EEP in very large prostates, we considered it necessary to review the studies exploring those cases. In this systematic review, we aimed to examine the efficacy and safety of laser-based EEP on prostate glands ≥150 ml.

## 2 Materials and methods

### 2.1 Search strategy

This systematic review registered with Research Registration Unique Identifying Number (UIN) of “reviewregistry1397” (https://www.researchregistry.com/). The study was carried out in accordance with the Preferred Reporting Items for Systematic Reviews and Meta-analyses protocols (PRISMA) statement [26]. A systematic search was conducted using Web of Science, PubMed-MEDLINE, Wiley Online Library and Cochrane Library databases until January 20, 2022 with the following search terms solely or in combination:"large prostate", "laser enucleation","laserprostatectomy". After retrieving the titles and abstracts of selected articles, the full texts of related articles were screened.

The objective of this systematic review was to assess the efficacy and safety of laser-based EEP in patients suffering symptomatic BPH with very large prostate concerning the following parameters: International Prostate Symptom Score (IPSS), maximum urinary fow rate (Qmax), post-void residual (PVR) and Quality of Life (QoL), intraoperative and postoperative complications (according to Clavien-Dindo classification) and re-treatment.

### 2.2 Eligibility criteria

**Fig. 1:**
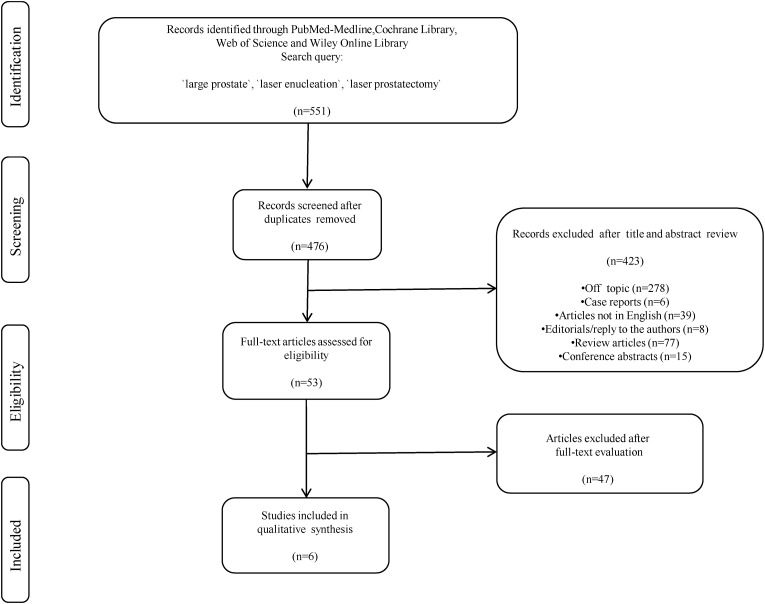
Preferred Reporting Items for Systematic Reviews and Meta-analyses (PRISMA) flow chart.

As proposed by the PRISMA guidelines, the PICO: Population (P), Intervention (I), Comparison (C), Outcomes (O) approach was taken to determine eligibility criteria [26]. The search strategy and article selection process are shown in PRISMA flow diagram (Fig. 1). We selected studies in which BPH patients (P) underwent laser enucleation of the prostate (I) specified prostate volumes more than 150 ml were compared (C) to assess urinary outcomes, perioperative complications, and safety (O). We excluded studies 1) unrelated to laser enucleation of the prostate, 2) without objectives or outcomes related to prostate volumes exceeding 150 ml, those that did not classify prostate volumes and/or failed to specify a specific prostate volume cut-off including >150 ml regarding surgical outcomes, 3) were not written in English, 4) review articles, editorials/letters, case reports, conference/meeting abstracts.

### 2.3 Data extraction

Articles relevant to our subject of interest were retrieved and evaluated independently by two authors (M.Y. and M.K.) and discrepancies were resolved through discussion by a third reviewer (T.T.). We documented the authors and date of study, study design, preoperative prostate volume (PV) stratifications, number of patients, mean ± SD or median (range) (IQR) values of age, preoperative and postoperative total serum PSA (ng/ml), IPSS or American Urological Association Symptom Score (AUASS), Qmax (ml/s), QoL, length of hospital stay (LOS) (days), operative time (OT) (min), duration of catheterization (DOC) (days), retreatment rate, 5α-Reductase inhibitors (5-ARI) and antiplatelet/anticoagulation (AP/AC) therapy status', enucleation (ET) and morcellation times (MT) (min), enucleation (EE) and morcellation efficiencies (ME) (g/min) and perioperative complications according to the Clavien-Dindo classification. Descriptive statistics were used for basic data, and a narrative synthesis was created.

### 2.4 Quality assessmentof the studies

Two reviewers (H.C.A. and S.T.) assessed each study independently using the Newcastle-Ottawa Scale (NOS) for non-randomised studies [27]. Reporting was performed based on the information contained in the relevant article. Any inconsistencies in the process of screening, identifying eligible articles, or risk assessments were discussed and resolved by consensus among reviewers prior to final reporting. The NOS consists of three subdomain: selection (maximum 4 stars/points), comparability (maximum 2 stars/points) and outcome (maximum 3 stars/points). Good quality studies achieve ≥6 stars/points. 4–6 stars/points and <4 stars/points represents fair quality and poor quality, respectively. Furthermore, we used the Assessment of Multiple Systematic Reviews (AMSTAR) 2 tool for the assessment of the quality of our systematic review [28].

## 3 Results

In searching through the aforementioned databases, we identified a total of 551 related studies. Overall, 75 duplicated studies were eliminated and 423 studies were excluded after title and abstract evaluation for the following reasons: inclusion criteria were not met or not related to laser enucleation of prostate, objective or outcome not related to mainly prostate volume (n = 278), not written in English (n = 39), case reports (n = 6), review articles (n = 77), conference abstracts (n = 15), editorials/reply to the authors (n = 8). After full-text evaluations, we excluded 47 more articles that had failed to classify by prostate volume or specify a prostate volume >150 ml as cut-off regarding surgical outcomes. Our final review included the remaining 6 publications [[18], [19], [20], [21], [22],^25^]. The 6 studies included 375 patients with prostate sizes >175 ml treated with laser-based EEP for symptomatic BPO. All studies in our study were retrospective. According to NOS, all included studies were judged to be of “good” quality. Furthermore, the quality of our systematic review was determined as “moderate”, with respect to AMSTAR 2 tool. In Table 1, we present the baseline characteristics and the methodological assessment of the studies our review includes. All six focused on HoLEP procedure outcomes. Among them, three studies examined HoLEP outcomes for prostate sizes >200 ml [[19], [20], [21]], two studies evaluated the HoLEP outcomes of patients with prostate glands larger than 175 ml [18,22], one of the studies evaluated the HoLEP surgical outcomes of patients with a PV of 200–299 and ≥ 300 ml [25]. Table 2 shows intraoperative variables and perioperative complications according to prostate volume and the Clavien-Dindo classification.

**Table 1:** Summary of demographics and baseline characteristics of the studies.

Author, year and study design	Procedure	Pre-op PV-TRUS or TAUS (ml)^a^	Nr. of patients	Age (years)^a^	PSA^a^ (ng/ml)	Pre-op IPSS^a^	Pre-op Qmax^a^ (ml/s)	Pre-op PVR (ml)	Pre-op QoL^a^	LOS (day)^a^	Post-op PSA^a^	Conversion to TURP or OP n (%)	DOC (day)^a^	Use of 5-ARI, n (%)	AP/AC therapy, n (%)	Re-treatment rate (%)	NOS quality score and quality
Boxall et al., 2021, retrospective	HoLEP	≥200: N/A	157	73 (67–77)	N/A	22 (18.5–26)	8.1 (6.3–10.5)	189.5 (120–254.8)	5 (4–5)	1 (1–2)	N/A	N/A	1 (1–1)	N/A	N/A	N/A	7- Good
Krambeck et al., 2010, retrospective	HoLEP	>175: 217.8 (174.6–391)	57	72 (48–90)	14.6 (1.6–48)	19 (2–30)^a^	8.2 (4.3–17.2)	N/A	N/A	26.5 (16–74) (hrs)	0.78 (0.28–1.5) (12th month)	1 (1.75)	1 (1–2)	N/A	N/A	0	8- Good
Assmus et al., 2021, retrospective	HoLEP	≥175: 229.9 (175–535)	55	73.8 (56–91)	8.58 (2.7–15.66)	22.3 (2–35)^a^	8.8 (2.7–19.5)	172 (37–600)	4.9 (2–6)	Same day discharge	0.87 (0.087–3.25) (3rd month)	N/A	Same day	N/A	0	0	7- Good
Zell et al., 2020, retrospective	HoLEP	200-299: 232.5 ± 27.5	76	73 ± 8	15 ± 12.2	18.7 ± 17.7	6.6 ± 4.4	N/A	N/A	1.7 ± 1.2	1.1 ± 1	2 (2.6)	2.5 ± 2.7	N/A	N/A	1.3	7- Good
		≥300: 385.5 ± 126.3	12	74.6 ± 5.9	18.8 ± 11.6	17.2 ± 8.7	6.5 ± 4.2			1.9 ± 1.2	1.5 ± 1	1 (8.3)	2.8 ± 2.9			0	8- Good
Glybochko et al., 2017, retrospective	HoLEP	>200: 230.1 ± 18.1	12	70.7 ± 5.1	N/A	19.5 ± 4.5	4.7 ± 0.9	72.3 ± 10.9	4.1 ± 0.3	3.3 ± 1.6	N/A	N/A	25.2 ± 2.4(hrs)	N/A	N/A	N/A	7- Good
Kim et al., 2015, retrospective	HoLEP	>200: 252.1 ± 59.5	6	72.7 ± 9.9	12.84 ± 3.22	13.2 ± 3.6	9.5 ± 3	118.7 ± 134.0	3.2 ± 1.2	3.5 ± 1.5	N/A	4 (66.6)	4.8 ± 4.8	N/A	2 (33.3)	0	7- Good

HoLEP: Holmium laser enucleation of the prostate; PV: prostate volume; PSA: prostate spesific antigen; IPSS: International Prostate Symptom Score; ICIQ-SF: International Consultation on Incontinence Questionnaire-Short Form; Qmax: maximum urinary flow rate; QoL: quality of life; LOS: Length of hospital stay; OT: operation time; SD: standard deviation; DOC: Duration of catheterization; OP: Open prostatectomy; TRUS: transurethral ultrasonography, TAUS: transabdominal ultrasonography; AP/AC: antiplatelet/anticoagulation; N/A: not applicable; NOS: Newcastle-Ottowa Scale quality assessment for nonrandomised studies.

^b^Δ: Change from baseline.

*Mean ± SD or median (range) (IQR).

a AUASS: American Urological Association Symptom Score.

**Table 2:** Intraoperative variables and perioperative complications according to patients’ prostate volume and Clavien-Dindo classification.

Author and year	PV (cc) *	ET (min) *	MT (min) *	EE (g/min) *	ME (g/min) *	OT (min) *	Clavien-Dindo classification, n(%)				
							I	II	III	IV	V
Boxall et al., 2021	≥200	70 (60–90)	45 (30–60)	2.8 (2.2–3.5)	4.4 (3.2–5.8)	N/A	N/A				
Krambeck et al., 2010	>175	91.9 (30–263)	38.6 (11–133)	N/A	N/A	N/A	N/A				
Assmus et al., 2021	≥175	72.5 (23–133)	23.3 (2–113)	N/A	N/A	121.6 (37–243)	5 (9)	7 (12.7)	0	1 (1.8)	0
Zell et al., 2020	200–299	77.4 ± 27.8	46.8 ± 30.2	2.±0.9	4.1 ± 2.5	170.7 ± 57	0	11 (14.8)	0	5 (6.75)	0
	≥300	83 ± 27	74.5 ± 31.1	2.6 ± 1.2	2.7 ± 1.4	182 ± 47.5	0	2 (16.6)	0	0	0
Glybochko et al., 2017	>200	120.9 ± 35	65.3 ± 13.2	1.70	3.16	N/A	N/A				
Kim et al., 2015	>200	76.7 ± 19.6	47.6 ± 28.2	1.49 ± 0.84	2.20 ± 0.69	N/A	N/A				

*Mean ± SD or median (range) (IQR).

**Abbreviations:** PV: prostate volume; ET: Enucleation time; MT: Morcellation time; EE: Enucleation efficiency; ME: Morcellation efficiency; OT: Operation time; N/A: not applicable.

Pooled postoperative outcomes of HoLEP surgery in patients with prostate volume >200 ml are illustrated from four studies in Table 3. Postoperative IPSS, Qmax, QoL, and PVR outcomes were pooled from two studies [19,20] at the 3-month follow-up, three studies [20,21,25] at 6-month follow-up, and two studies [20,25] at 12-month follow-up.

**Table 3:** Pooled postoperative outcomes of patients with a prostate volume >200 ml.

	Postop IPSS	ΔIPSS	Postop Qmax (ml/sec)	ΔQmax (ml/sec)	Postop QoL	ΔQoL	Postop PVR (ml)	ΔPVR (ml)
**At 1.Month** (n = 12)	5.5 ± 1.0	–14 ± 1.24	21.3 ± 1.7	+16.6 ± 5.89	1.7 ± 0.9	–2.4 ± 1.22	14.0 ± 10.1	–58.3 ± 5.4
Glybochko et al., 2017								
**At 3. Month** (n = 169)	5.04 ± 1.94	–18.16 ± 4.4	19.58 ± 6.44	+11.73 ± 0.43	1.04 ± 0.42	–3.89 ± 2.29	28.54 ± 35.13	–152.63 ± 144
Boxall et al., 2021								
Glybochko et al., 2017								
**At 6. Month** (n = 94)	6.49 ± 4.86	–11.95 ± 1.48	21.83 ± 14.99	+15.29 ± 0.42	2.2 ± 1.82	N/A	34.8 ± 42.85	N/A
Glybochko et al., 2017								
Kim et al., 2015								
Zell et al., 2020,								
**At 12. Month** (n = 88)	4.9 ± 4.47	–13.9 ± 0.99	21.33 ± 9.9	+14.99 ± 0.5	2.07 ± 1.75	N/A	16.6 ± 8.5	N/A
Zell et al., 2020								
Glybochko et al., 2017								
**At 18. Month** (n = 12)	5.5 ± 1.5	–14 ± 1.42	25.0 ± 1.8	+20.3 ± 6.51	1.9 ± 0.7	–2.2 ± 1.63	16.1 ± 6.5	–56.2 ± 3.45
Glybochko et al., 2017								

IPSS: International Prostate Symptom Score; Qmax: maximum urinary flow rate; PVR: postvoiding residual volume; QoL: quality of life.

### 3.1 Perioperative and urinary outcomes

Table 4 presents the postoperative outcomes of all studies included in our review. Boxall et al. [19] likewise investigated HoLEP's efficacy and safety in 314 male patients with very large prostates. In their retrospective study, the patients were divided into two groups as 80–199 and ≥200 ml according to prostate volume. They found enucleation and morcellation times were significantly higher in the group with PV ≥ 200 ml (p < 0.001). No significant difference was observed between their groups in terms of Qmax [19.3 (14.5–27.8) vs. 19.2 (16.8–26.1); p = 0.915], IPSS [5 (3–7) vs. 4 (3–8); p = 0.737], QoL [1 (0–2) vs. 1 (0–2); p = 0.538], PVR [29.5 (0–75.5) ml vs. 20 (0–64); p = 0.559] in the 3rd month postoperative follow-up.

**Table 4:** Postoperative outcomes of patients according to prostate volumes.

	PV (ml)	Post-op IPSS	Post-op Qmax (ml/sec)	Post-op QoL	Post-op PVR (ml)
**At 1. Month**					
Glybochko et al., 2017 (n = 12)	>200	5.5 ± 1.0	21.3 ± 1.7	1.7 ± 0.9	14.0 ± 10.1
**At 3. month**					
Boxall et al., 2021 (n = 157)	≥200	5 (3–7)	19.3 (14.5–27.8)	1 (0–2)	29.5 (0–75.5)
Glybochko et al., 2017 (n = 12)	>200	5.7 ± 1.1	23.3 ± 1.5	1.7 ± 0.5	16.1 ± 8.8
Assmus et al., 2021 (n = 55)	≥175	6.7 (2–12)*	20.4 (10.9–29.9)	1.3 (0–2)	25 (0–109)
**At 6. month**					
Krambeck et al., 2010 (n = 57)	>175	6.5(1–28)*	18.5(4.3–31.5)	N/A	N/A
Glybochko et al., 2017 (n = 12)	>200	5.5 ± 1.2	23.9 ± 1.9	1.7 ± 0.4	16.7 ± 8.6
Kim et al., 2015 (n = 6)	>200	3.3 ± 2.2	20.7 ± 6.1	0.8 ± 1.6	71.0 ± 80.3
Zell et al., 2020 (n = 82)	200–299	6.9 ± 5.4	21.6 ± 16.7	2.4 ± 2.0	N/A
	≥300	6.0 ± 5.8	19.1 ± 15	2.5 ± 2.8	N/A
**At 12. month**					
Krambeck et al., 2010 (n = 57)	>175	4.3 (0.11–12.2)*	12.2 (7.7–16.7)	N/A	N/A
Zell et al., 2020 (n = 81)	200–299	4.8 ± 4.8	20.9 ± 10.	2.1 ± 1.9	N/A
	≥300	3.8 ± 3.3	25.4 ± 20.6	0.6 ± 0.9	N/A
Glybochko et al., 2017 (n = 12)	>200	5.6 ± 1.7	24.1 ± 2.2	1.9 ± 0.4	16.6 ± 8.5
**At 18. month**					
Glybochko et al., 2017 (n = 12)	>200	5.5 ± 1.5	25.0 ± 1.8	1.9 ± 0.7	16.1 ± 6.5

IPSS: International Prostate Symptom Score; Qmax: maximum urinary flow rate; PVR: postvoiding residual volume; QoL: quality of life.

In a retrospective study, Krambeck et al. [22] described surgical outcomes of HoLEP in patients with a PV > 175 ml. Mean catheterization time and LOS were 18.5 and 26.5 h, respectively. They reported that the AUASS fell by 65.8% and 77.3% compared to preoperative baseline values, and a rise by 55.7% and 33.8% in Qmax at the 6th and 12th month follow-ups. Similarly, Assmus et al. retrospectively evaluated the same-day discharge and functional outcomes of patients with a prostate gland ≥175 ml who underwent HoLEP [18]. 55 patients were included in their study, 82% of whom were discharged on the same day and whose catheterization time was 21.2 h. They observed a statistically significant improvement in AUASS, QoL and PVR compared to baseline values at the 3rd month follow-up (p < 0.001, p < 0.001 and p = 0.003, respectively). Qmax increased statistically significantly from 8.8 to 20.4 at the 3rd month follow-up (p = 0.032).

Zell et al. retrospectively evaluated the HoLEP outcomes of patients with a prostate size exceeding 200 ml [25]. According to PV, patients were divided into two groups as 200–299 ml (n = 76) and >300 ml (n = 12). While there was no statistical difference between these two groups in ET; MT was statistically significantly longer in the group with PV > 300 ml (p = 0.38, p = 0.02; respectively). Mean LOS was 1.8 ± 1.2 days and the mean catheterization time was 2.6 ± 2.7 days. They reported no statistically significant difference between these two groups in mean LOS and catatherization times. In terms of functional outcomes, at the 12th month follow-up, the mean IPSS fell from 18.7 to 4.8 in the group with PV 200–299 ml and from 17.2 to 3.8 in the group with PV > 300 ml. The mean Qmax increased from 6.6 to 20.9 in the group with PV 200–299 ml and from 6.5 to 25.4 in the group with PV > 300 ml group. However, there was no statistical difference in the mean IPSS, Qmax and QoL between the two groups at their 12th month follow-up.

Glybochko et al. retrospectively investigated HoLEP outcomes in patients with various prostate gland sizes [20]. Patients were divided into 3 groups (G) according to their prostate volume as <100 ml (n = 278) (G1), 100–200 ml (n = 169) (G2) and >200 ml (n = 12) (G3). They observed that the mean ET and MT rose in conjunction with the prostate size (Mean ET: 56.5 ± 10.7 min, 96.4 ± 24.9 min, 120.9 ± 35, Mean MT: 27.5 ± 7.3 min, 43.3 ± 11.2 min, 65.3 ± 13.2 min, for the groups; respectively). The mean ET (for G1-3 p < 0.001, for G2-3 p = 0.03) and MT (for G1-3 p < 0.001, for G2-3 p = 0.03) were statistically significantly longer in the group with a PV of >200 ml. The duration of catheterization was 24 h. LOS lasted approximately 3 days in all three groups. There was no statistically significant difference among three groups in terms of IPSS, Qmax, QoL and PVR at 18-month follow-ups (p > 0.05).

In their retrospective study, Kim et al. divided patients into 3 groups according to prostate volume as <100 ml (n = 426), 100–200 ml (n = 70) and >200 ml (n = 6) [21]. They found mean ET and MT to be statistically significantly longer in the group with PV > 200 ml, similar to the Glybochko et al. study (p < 0.001). Mean LOS and catheterization time were longer in the group with a PV of >200 ml (p = 0.011 and p = 0.004, respectively). IPSS, QoL and Qmax improvements at 6th months were statistically significant in all 3 groups (IPSS: p < 0.001, p < 0.001, p = 0.002, QoL: p < 0.001, p < 0.001, p = 0.022, Qmax: p < 0.001, p < 0.001, p = 0.023, respectively).

### 3.2 Intraoperative and postoperative complications

Boxall et al. did not classify complications according to the Clavien-Dindo classification [19]. In contrast, they compared blood transfusion needs in 2 groups with PV of 80–199 and > 200 ml (1.5% vs 4.5%). They detected no statistical difference between groups in terms of transfusion requirements, and observed urinary incontinence (UI) requiring the use of a pad in 26 (8.3%) patients at 3rd months postoperatively. UI was higher in patients with a PV of ≥200 ml (n = 17, 10.8%) than in the 80–199 ml group (n = 9, 5.8%), but this difference was not statistically significant.

Krambeck et al. reported 3 complications in their study that included 57 patients with a PV > 175 ml [22]. One patient suffered a postoperative urethral stricture. Morcellation was impossible in only 1 patient because of the density of a prostate adenoma, for which a cystostomy was performed to remove tissue. No persisting UI was observed up to 12th months postoperatively [22]. Assmus et al. reported postoperative complications in 13 (23.6%) patients with a PV ≥ 175 ml [18]. 12 (21.8%) of the complications were Clavien-Dindo grade I-II. Fever and hematuria occurred in only one patient after discharge (Clavien-Dindo IVa). This patient was hospitalized because of urosepsis and acute kidney failure.

In the study of Zell et al., perioperative and postoperative complication rates (22.4% vs 16.7%) were higher in the group with a PV of 200–299 ml than in the >300 ml group, but this difference was not statistically significant [25]. Similarly, the transfusion rate was higher in the group with a PV of 200–299 ml but that was not statistically significant either (8.3% vs 21.6%, p > 0.05). In total, 5 patients developed Clavien-Dindo grade IVa complications - all of which occurred in the group with PV 200–299 ml. Conversion to open prostatectomy was required in 3 (3.4%) patients during the procedure, 2 of these were in the group with a PV of 200–299 ml (p > 0.05).They reported postoperative UI in only three patients (3.4%) in their PV > 200 ml group [25].On the other hand, in the study by Glybochko et al., transient UI was observed in 16.7% of patients with a PV of >200 ml [20].

Kim et al. reported that one patient with a PV of >200 ml suffered recurring urethral strictures, although no surgical treatment was required [21]. Transient urge UI was found to be statistically significantly higher in 2 patients (33.3%) in their PV > 200 ml group (p = 0.015).

### 3.3 Re-treatment

Krambeck et al. reported no need of re-treatment in patients with a PV of >175 ml [22]. In the study by Assmus et al., second surgery was not required in patients with a PV of ≥175 ml at 3-month follow-up [18]. In the study of Zell et al., one patient (1.1%) with a PV of 200–299 ml required second HoLEP at 43.5-month follow-up [25]. However, no patients in groups with a PV of ≥300 ml required re-treatment. In the Kim et al.study, there was no need for re-intervention at the 6-month follow-up [21].

## 4 Discussion

Surgical options may be considered to be limited in very large prostates, particularily if the procedure is to be performed transurethrally. SP has historically been considered the ideal surgical method for large prostates. In light of the latest evidence, this paradigm is evolving toward minimally-invasive endoscopic solutions. EAU guidelines on the surgical treatment of large prostates recommend SP for large prostates (>80 ml) when endoscopic prostate enucleation is not available [12]. Except for prostate sizes of 80–100 ml, there are very few studies reporting 150, 175 and 200 ml as a cut off value to investigate the efficiency and safety of laser enucleation in prostates this large. Apart from the large glands mentioned in the guidelines, there is no consensus as to which surgical method is ideal in patients with much larger prostates. In the literature, the giant or “mega” prostate-size definition tends to be applied for prostate volumes of 200 ml and over [19,20]. In addition, prostates exceeding 150 ml in the AUA BPH guideline are classified as ‘very large prostates' [29]. We therefore considered 150 ml as the lower-volume limit as being appropriate for defining a ‘very large prostate’, and we chose to include prostates of ≥150 ml up in our search strategy. The studies we reviewed showed that laser enucleation is an effective, safe and feasible surgical method in very large and giant prostates.

Tecnological advances in technology have not just popularized the use of lasers in urology, but also laparoscopic and robot-assisted surgeries. Laparoscopic simple prostatectomy (LSP) and robot-assisted simple prostatectomy (RASP) in large prostates have thus become more widespread. In a recent prospective multicenter randomized study, Fuschi et al.compared HoLEP with other minimally invasive approaches (LSP and RASP) in prostates ≥120 ml [30]; they detected no difference between groups in terms of functional and perioperative results, except that the catheterization duration was statistically significantly longer in the LSP group (P = 0.002). They also found that LOS lasted longer in the LSP and RASP groups than the HoLEP group [30]. In another study, SP, RASP and Thulium laser vapoenucleation (ThuVEP) were compared in patients with prostates >80 ml, and no difference was observed between surgical approaches in postoperative urine flow. Operation time (OT), blood loss and blood transfusion were all less in the laser group compared to SP and RASP (p < 0.05, p < 0.01, p > 0.05; respectively). Urinary incontinence was observed significantly more often in the SP group than in the minimally invasive groups (p ≤ 0.001) [31]. These two studies show that although functional results are similar, patients who underwent laser-based EEP especially HoLEP seemed to benefit more in terms of hospitalization and catheterization times. SP is also associated with higher rates of prolonged recovery and postoperative pain [5].

In another study, Gunseren et al. investigated the effect of prostate size on operative time in patients with >80 ml prostate underwent HoLEP, SP, and LSP [32], and found that the OT and prostate removal rate were better in the HoLEP group than the LSP group (89.6 ± 27.4 min vs. 124.8 ± 40.2 min, respectively, p = 0.000 and 1.08 ± 0.23 g/min vs. 0.90 ± 0.32 g/min, respectively, p = 0.000). LOS(1 ± 0.1 vs. 6.1 ± 3.4 vs. 7.5 ± 3.6 days, respectively; p = 0.000) and catheterization times (3 ± 0.3 vs. 6.4 ± 0.8 vs. 8.8 ± 2.7 days, respectively; p = 0.000) were found to be shorter in the HoLEP group. Interestingly, they noted a significant correlation between prostate size and OT only in their HoLEP group (p = 0.000; R = 0.743), and OT of prostates >110 g was higher in HoLEP than SP. Similarly, in the studies we reviewed, we observed that the OT becomes longer as the prostate size increases. OT stands out as an important perioperative parameter to be considered during laser surgery in patients with very large-giant prostates. Due to prolonged ET and MT, disorientation during the procedure and extra effort to ensure bleeding control, operation times for very large and giant prostates may be prolonged. Surgeons need to keep in mind that as prolonged general anesthesia is risky, operation times must never be disregarded [33].

We observed better postoperative functional outcomes (IPSS, PVR, and Qmax) in prostates with a volume of ≥175, >200 and >300 ml in the present study, and no difference in postoperative functional results between patient groups with prostate sizes of 100–200 ml, > 200 ml or even >300 ml. In addition, we observed improved functional parameters, especially when analyzing the pooled data including studies that included prostates exceeding 200 ml (Table 3). These findings support that, in terms of efficacy, laser enucleation of the prostate is feasible regardless of its size. Furthermore, we detected no significant difference between 100 and 199 and > 200 and >300 ml prostate sizes regarding perioperative and postoperative complications. Most of the complications were Clavien-Dindo I (%0–9) and II (%12.7–16.6), postoperative hematuria, dysuria, urinary tract infection. Postoperative urethral stricture was observed in one patient (%1.7) with a prostate of >175 ml in the study by Krambeck et al. and in one patient (%16.7) with a prostate of >200 ml in the study by Kim et al. [21,22]. Note that increasing the duration and intensity of the mechanical maneuvers required to enucleate-giant prostates may raise the risk of urethral mucosal trauma leading to urethral stricture. In terms of postoperative incontinence, we observed that urinary incontinence rates varied between 3.4 and 33.3% in patient groups with prostates measuring >200 ml. Kim et al. found that 33.3% (n = 2) of their >200 ml prostate group suffered from transient urge urinary incontinence [21]. We emphasize that the preoperative incontinence status and incontinence type of patients included in the studies we reviewed was not specified. The incontinence rate differences between studies may be attributable to differences in preoperative continence status, surgical techniques, and divergent surgical experience.

When an adenoma is excessively large, it may not be completely removable, and reoperation may eventually become necessary. The retreatment rate was 0–1.3% in all studies involving prostate size ≥175 ml in this study. In the study with the longest follow-up (43.5 months), Zell et al. stated that while no patient with a PV of >300 ml required reoperation, one patient whose prostate measured 200–300 ml needed retreatment [25]. Reoperation rates after LEP for giant prostates are acceptable.

## 5 Operative challenges and recommendations for very large prostates

Larger prostates are more likely to present high vascular density [21], thus their removal can cause bleeding and clot retention. Bleeding compromises the endoscopic view, making the morcellation step hazardous [20]. Effective bleeding control before morcellation is therefore vital for patient safety. In addition to enabling hemostasis via laser use, coagulating the prostatic fossa with a cutter loop or roller probe after enucleation is also recommended before morcellation [34]. Furthermore, multiple hyperplastic nodules are common in huge prostates [20,21,25]. These nodules may obscure the sugeon's view of the surgical capsule and plane, thus making enucleation harder. In this case, firstly, the main adenoma is enucleated by following a carefully established surgical plan, after which the satellite adenoma can be enucleated [21]. Uncertainty that may arise through the surgical plan can thus be alleviated.

It is generally harder to morcellate very large prostates than small to moderate ones. Some authors maintain that perineal urethrostomy/cystostomy may be necessary to remove large adenomas [[18], [19], [20],^22^]. In patients whose enucleated giant adenoma leaves no safe area for morcellation in the bladder, or if the surgical instruments are too short to access the giant adenoma, cystostomy may be preferable [19,20]. Boxall et al. reported that adenoma nodules, which they define as “beach-balls”, are common in giant prostates, and the morcellator blades have trouble cutting such rubber-like nodules [19]. We recommend removing them with the help of a stone grasper. If hard adenoma tissue is encountered, transurethral resection may be required for tissue retrieval [21].

One of the main challenges associated with very large prostates is maintaining the proper orientation during surgery. Since it is harder to determine anatomical landmarks in giant prostates than in those of small to moderate size, keeping one's orientation might take too much time and thus prolong the cumulative surgery duration [35]. Another problem in the removal of very large prostates is that our surgical instruments are not long enough, simply because the entire urethra may be too long. This factor can complicate the enucleation, especially around the bladder neck [21]. Moreover, large prostates may also compromise the surgical instrument's manipulation. Since voluminous prostates may protrude into the bladder, ureteral orifices may be injured during endoscopic manipulation. Surgeons therefore need to keep the distance between the ureteral orifices and adenoma in mind when enucleating around the bladder neck [20].

To the best of our knowledge, this is the first systematic review focusing on LEP procedures in very large prostates. However, the present study has some limitations to acknowledge. All the studies reviewed were retrospective, and there was wide variation in participants numbers making the data heterogeneous. Furthermore, as there is a paucity of published investigations on very large and giant prostates, the articles reviewed in this study are few.

## 6 Conclusions

Laser-based-EEP is an efficient, safe and feasible procedure even in very large glands enabling improved functional outcomes as well as low perioperative complication and retreatment rates. More high-quality studies with larger patient cohorts are needed to better demonstrate the advantages of laser-based EEP in very large prostates. Special surgical devices, particularily longer endoscopes, might be required to make enucleation of such glands easier.

## Ethical approval

As this study is a narrative review, the ethical approval is not required.

## Sources of funding

The authors declare no funding received.

## Author contribution

M.Y. and M.K. conceived the study concept and design, performed literature search and wrote the manuscript. H.C.A. and T.T. interpreted the data. M.F.B., S.T., C.G. and A.M. provided critical review. A.M. supervised the manuscript. All authors discussed the results and commented on the manuscript. All authors read and approved the final manuscript.

## Consent

This article does not contain any studies with human participants or animals performed by any of the authors.

## Registration of research studies

1. Name of the registry:
2. Unique Identifying number or registration ID:
3. Hyperlink to your specific registration (must be publicly accessible and will be checked):

## Guarantor

Arkadiusz Miernik, MD, PhD, FEBU, MHBA Professor of Urology University of Freiburg – Medical Centre, Faculty of Medicine Department of Urology Hugstetter Str. 55, D-79106 Freiburg, Germany.

## Research involving human participants and/or animals

This article does not contain any studies with human participants or animals performed by any of the authors.

## Data availability statement

The data analyzed during the current study are available from the corresponding author on reasonable request.

## Provenance and peer review

Not commissioned, externally peer-reviewed.

## Declaration of competing interest

A. Miernik receives research funds of the German 10.13039/501100002347Federal Ministry of Education and Research, Berlin (D). He receives support for his travel activities from the European Society of Urology, Arnhem (NL), and the 10.13039/501100006186German Society of Urology, Düsseldorf (D). Furthermore, A. Miernik is consulted for: KLS Martin, Tuttlingen (D), Avateramedical, Jena (D), LISA LaserProducts GmbH, Katlenburg-Lindau (D), Schoellyfiberoptics GmbH, Denzlingen (D), Dornier MedTech Laser GmbH (D), Medi-Tate Ltd. (IL, USA) and B. BraunNewventures GmbH, Freiburg (D). A. Miernik is speaker for the companies Richard Wolf GmbH (D) and Boston Scientific (USA). Additionally, he performed expert activities for the Ludwig Boltzmann Gesellschaft, Wien (A). A. Miernik is involved in numerous patents and inventions in the field of medical technology. C. Gratzke is advisor for Astellas Pharma GmbH, Munich (D), Ipsen Pharma GmbH, Munich (D), Steba Biotech S.A., Luxembourg (LUX), Bayer Pharma, Leverkusen (D), Olympus Winter &Ibe GmbH, Hamburg (D), Medi-Tate Ltd., Or Akiva (IL), MSD, Haar (D), Astra-Zeneca, Cambridge (UK) and Roche, Basel (CH). C. Gratzke receives speaker fees from 10.13039/100002429Amgen, California (10.13039/100011408USA), 10.13039/501100004948Astellas Pharma GmbH, Munich (D), 10.13039/501100014382Ipsen Pharma GmbH, Munich (D), 10.13039/100015756Janssen-Cilag GmbH, Neuss (D), 10.13039/100004326Bayer Pharma, Leverkusen (D), Takeda Pharmaceuticals, Tokio (JPN) and 10.13039/501100014841medac GmbH, Wedel (D). MY, MK, HCA, MFB, ST, and TT declare to have no conflicts of interest.
